# Molecular profiling and specific targeting of gemcitabine-resistant subclones in heterogeneous pancreatic cancer cell populations

**DOI:** 10.3389/fonc.2023.1230382

**Published:** 2023-08-31

**Authors:** Benedikt Färber, Olga Lapshyna, Axel Künstner, Michael Kohl, Thorben Sauer, Kira Bichmann, Benjamin Heckelmann, Jessica Watzelt, Kim Honselmann, Louisa Bolm, Meike ten Winkel, Hauke Busch, Hendrik Ungefroren, Tobias Keck, Timo Gemoll, Ulrich F. Wellner, Rüdiger Braun

**Affiliations:** ^1^ Department of Surgery, University Medical Center Schleswig-Holstein, Lübeck, Germany; ^2^ Medical Systems Biology Group, Lübeck Institute of Experimental Dermatology, University of Lübeck, Lübeck, Germany; ^3^ Institute for Cardiogenetics, University of Lübeck, Lübeck, Germany; ^4^ Section for Translational Surgical Oncology & Biobanking, Department of Surgery, University Hospital Schleswig-Holstein, University of Lübeck, Lübeck, Germany; ^5^ First Department of Medicine, University Medical Center Schleswig-Holstein, Lübeck, Germany; ^6^ Institute of Pathology, University Medical Center Schleswig-Holstein, Kiel, Germany

**Keywords:** pancreatic cancer, intratumor heterogeneity, treatment response, gemcitabine, chemotherapy

## Abstract

**Purpose:**

Chemotherapy is pivotal in the multimodal treatment of pancreatic ductal adenocarcinoma (PDAC). Technical advances unveiled a high degree of inter- and intratumoral heterogeneity. We hypothesized that intratumoral heterogeneity (ITH) impacts response to gemcitabine treatment and demands specific targeting of resistant subclones.

**Methods:**

Using single cell-derived cell lines (SCDCLs) from the classical cell line BxPC3 and the basal-like cell line Panc-1, we addressed the effect of ITH on response to gemcitabine treatment.

**Results:**

Individual SCDCLs of both parental tumor cell populations showed considerable heterogeneity in response to gemcitabine. Unsupervised PCA including the 1,000 most variably expressed genes showed a clustering of the SCDCLs according to their respective sensitivity to gemcitabine treatment for BxPC3, while this was less clear for Panc-1. In BxPC3 SCDCLs, enriched signaling pathways EMT, TNF signaling via NfKB, and IL2STAT5 signaling correlated with more resistant behavior to gemcitabine. In Panc-1 SCDCLs MYC targets V1 and V2 as well as E2F targets were associated with stronger resistance. We used recursive feature elimination for Feature Selection in order to compute sets of proteins that showed strong association with the response to gemcitabine. The optimal protein set calculated for Panc-1 comprised fewer proteins in comparison to the protein set determined for BxPC3. Based on molecular profiles, we could show that the gemcitabine-resistant SCDCLs of both BxPC3 and Panc-1 are more sensitive to the BET inhibitor JQ1 compared to the respective gemcitabine-sensitive SCDCLs.

**Conclusion:**

Our model system of SCDCLs identified gemcitabine-resistant subclones and provides evidence for the critical role of ITH for treatment response in PDAC. We exploited molecular differences as the basis for differential response and used these for more targeted therapy of resistant subclones.

## Introduction

Pancreatic cancer is one of the most aggressive and lethal cancers worldwide, and the fourth leading cause of cancer-associated deaths (1). It has been predicted that pancreatic cancer will be the second most common cancer-related cause of death by 2030 in the United States (2). Surgical therapy is currently the only curative treatment option, but only about 20% of patients are eligible for this treatment option at the time of diagnosis (3). This is followed by adjuvant chemotherapy, which prolongs the median overall survival of patients, depending on the chemotherapy regimen to 35.0 and 54.4 months for gemcitabine and modified FOLFIRINOX, respectively (4). In patients with metastatic pancreatic cancer, the administration of FOLFIRINOX leads to a survival advantage with a median overall survival of 11.1 months compared to 6.8 months under gemcitabine treatment (5). However, the choice of chemotherapeutic agents is based on the patient’s physical condition, while the individual biology of the tumor, unlike in other cancer entities, has not played a role in clinical routine practice so far.

In recent years, several studies have suggested the classification of pancreatic ductal adenocarcinomas (PDACs) into different subgroups based on their molecular signature (6–8). Currently, one of the most commonly used classification system is based on transcriptomic subtypes, e.g., the subdivision by Moffit et al. into a classical type and a more aggressive basal-like type (8). Those assignments indeed correlate with patient overall survival and likewise with a certain resistance or sensitivity against specific chemotherapies, but the correlation of the overall survival rate only applies to early stages (9).

At the single-cell level, it became evident that tumor cells of both subtypes coexist within one tumor. The entirety of these co-existing subpopulations make up the expression profile of the tumor mass. It can therefore be concluded that the genomic and transcriptomic profiles are determined by a continuum of gene expressions derived from a mixture of subpopulations within a pancreatic tumor (9, 10). This intratumoral heterogeneity (ITH) is hard to capture sufficiently by bulk analyses (11).

ITH has become apparent to play an important role in tumor biology and thus also determines the response to the selected therapy options and ultimately overall survival as shown in various tumor entities (12). Genomic instability causes the tumor cells to generate numerous genetic changes and a branching evolutionary process of tumor clones is created (13). Most of these changes do not benefit the subclones and an equilibrium in the context of a functional hierarchy is created in the tumor cell population (14). This functional heterogeneity is also reflected in differences in intrinsic sensitivity to specific drugs and external changes, such as chemotherapy, can disturb this balance and give certain subclones a selection advantage, which is also reflected in tumor recurrence (15, 16). Our previous studies in Panc-1 cells have shown phenotypic and functional heterogeneity, i.e., with respect to epithelial-mesenchymal transition (EMT), stem cell marker expression and response to growth factors (17).

In our present study, we pursue the hypothesis that ITH influences the treatment response of PDAC since resistant subclones are already present within the tumor cell population before treatment. By establishing single cell-derived cell lines (SCDCLs) of the classical cell line BxPC3 and the basal-like cell line Panc-1, we aim to uncover heterogeneity of treatment response of distinct tumor cell subpopulations in an *in vitro* model. Subsequently, we aim to uncover the molecular preconditions of tumor cell subclones that correlate with their distinct response to therapy using transcriptomic and proteomic profiling. Understanding subclonal resistance mechanisms in heterogeneous tumor cell populations might ultimately help to develop new clinical treatment strategies in PDAC.

## Materials and methods

### Cell culture and establishment of SCDCLs

The PDAC-derived cell line BxPC3 (classical subtype) and Panc-1 (basal-like subtype) were cultured in DMEM high glucose with 10% fetal bovine serum and 1% Penicillin-Streptomycin-Glutamine (Sigma-Aldrich, St. Louis, USA) at 37°C, 5% CO_2_ in a humidified atmosphere. Mycoplasma contamination was excluded in both cell lines by PCR (MycoScope PCR Detection Kit, Genlantis, San Diego, CA). Cell line authentication was performed by short tandem repeat (STR) profiling using the PowerPlex^®^ 21 System (Promega, Madison, USA) according to the manufacturer’s instructions.

To generate single cell-derived cell lines (SCDCLs), limited dilution of both parental cell lines was performed in 96-well plates. For Panc-1 two and for BxPC3 four 96-well plates were seeded initially. Each well was examined by phase-contrast microscopy three hours after plating to ensure that only wells harboring a single cell were used for further cultivation. Once the single-cell clones reached approximately 80% confluency in a 96-well plate, they were transferred to a 6-well plate. Upon reaching 80% confulency in the 6-well-plate, the SCDCLs were further transferred to a T-25 flask. After reaching 80% confluency in the T-25 flask, both RNA and protein samples were collected.

### Treatment response of individual SCDCLs to gemcitabine

To measure the sensitivity to gemcitabine (Sigma-Aldrich, St. Louis, USA), cells of the respective parental cell population growing in log phase (2,000 cells of BxPC3 and 1,500 cells of Panc-1 per well) were seeded in 96-well plates. After a period of 24 hours, the cells were treated with gemcitabine concentrations ranging from 2.5 to 320 nmol/L for 72 hours. After this time, the survival fraction of the cells was determined. For this purpose, cell metabolism was used as a surrogate parameter for viability by using the CellTiter-Blue assay (Promega, Madison, USA). Normalization was based on untreated controls. IC_50_ and IC_max_ of parental cell populations were determined using dose-response curves. Subsequently, each SCDCL was treated with gemcitabine in the same experimental set-up at the IC_50_ and IC_max_ of the respective parental cell population. Finally, for selected SCDCLs, complete dose-response curves were generated, as performed for the parental cell lines. Each individual measurement was carried out in triplicates and repeated three times.

### Proliferation curves

Cells were seeded at 20,000 cells per well in a 6-well plate(Sarstedt AG & Co. KG, Nümbrecht, Deutschland). Cells were detached from a well by trypsination and counted with a Neubauer chamber (Brand GmbH & Co. KG, Wertheim, Deutschland) every 24 hours. Counting results were normalized to day 1. For each proliferation curve, three independent biological replicates were performed for each time point.

### Total mRNA sequencing

RNA was extracted from each SCDCL using the AllPrep RNeasy Mini Kit (Qiagen N.V., Venlo, Niederlande) as indicated by the manufacturer in the instruction manual. RNA samples were sequenced at Novogene Europe, Cambridge, United Kingdom. A poly-A enrichment and strand-specific library preparation were used. Sequencing was performed on an Illumina Novaseq6000 with S4 flowcell and PE150 length aiming for 30 million reads per sample. The data have been deposited to GEO Accession with the data set identifier GSE232549.

### Pathway and gene set analyses

Raw sequencing data (fastq format) was mapped against the human transcriptome (Ensembl GRCh38.103) using kallisto (v0.46.1) and differential expression analysis was performed using sleuth (v0.30.0) (18, 19). Gene set enrichment analysis (GSEA) on b-values (effect sizes estimated by sleuth) was performed using mitch (v1.4.1) against HALLMARK gene sets extracted from the msigdf R package (v7.0) (20).

### Label-free micro-LC tandem mass spectrometry

Protein from each SCDCL was extracted using the EasyPep™ Mini MS Sample Prep Kit (Thermo Fisher Scientific Inc., Waltham, USA). Extracted protein was analyzed using label-free micro-LC tandem mass spectrometry (Ultimate 3000 nHPLC, ThermoFisher & 5600+ Triple TOF, AB Sciex) using data-independent acquisition (DIA). After digestion of non‐labeled protein samples with trypsin, transmitted ions were fragmented and analyzed in the TOF MS Analyzer at high resolution. The raw SWATH data were processed using the software tool DIA-NN v1.7.16 (data-independent acquisition by neural networks) developed by Vadim Demichev et al. (21). The software was used in the high accuracy LC mode with RT-dependent cross-normalization enabled. Mass accuracy, MS1 accuracy, and scan window settings were set to 0, as DIA-NN optimizes these parameters automatically. The ‘match between runs’ function was used to first develop a spectral library using the ‘smart profiling strategy’ from the data-independent acquisition data. The human UniProtKB/swiss-prot database (version 2020/12/6) was used for protein inference from identified peptides (22). Trypsin/P was specified as protease. The precursor ion generation settings were set to peptide length of 7–52 amino acids, the maximum number of missed cleavages to one. The maximum number of variable modifications was set to zero. N-terminal methionine excision and cysteine carbamidomethylation were enabled as fixed modifications. The resulting report file was further processed in the DIA-NN R package for MaxLFQ-based protein quantification (21, 23). A report was generated containing unique proteins (proteins that were not assigned to a group of homologs) that passed the FDR cut-off of 0.01 applied on the precursor level and were identified and quantified using proteotypic peptides only.

The mass spectrometry proteomics data have been deposited to the ProteomeXchange Consortium via the PRIDE partner repository with the dataset identifier PXD042256 (24, 25).

### Proteomic feature extraction for treatment response

For calculation of the most important proteins that were related to the heterogeneity of SCDCLs we used the R software (v. 4.1.2) along with the packages caret and klaR (26–28). Both packages are available from the CRAN.repository (https://cran.r-project.org/). Using the random forest (RF) algorithm implemented in the caret package the protein data obtained from the MS experiments were used to fit a regression model. Measured response to the IC_50_ value of gemcitabine of the respective parental tumor population was used as target variable of the model. ‘Backward Feature Elimination’ was utilized to select the most important proteins for the best fit of the regression model. To this end, the ‘Root Mean Square Error’ (RMSE) served as performance measure and the protein set that yielded the best RMSE value was selected from each model run. Using different seed values we took advantage of the random characteristics of the RF algorithm and performed several replicates for both cell lines (10 for Panc-; 30 replicates for BxPC3) of the model runs. In our model, a higher ranking of a protein within a list of the respective run corresponded to a higher relevance for explaining heterogeneous therapy response to IC_50_ of gemcitabine of the respective parental tumor population. As each run computed an at least slightly different set of ‘optimal’ proteins the results of all model runs were integrated by calculating a total score for each protein as follows: $\sum_{k=1}^{n}Xk=1-\frac{a-1}{b}$ (b=“number of proteins in a run”, a=“rank position of the protein in this run”, n=total number of runs). We arbitrarily chose 50 as the upper limit for the number of proteins in the final list because the average number of proteins determined for BxPC3 corresponded to this order of magnitude, whereas the number of proteins determined for Panc-1 was significantly lower.

### Treatment response of individual SCDCLs to JQ1

To measure the sensitivity to JQ1 (APExBIO, Houston, USA), cells of the respective parental cell population or SCDCLs growing in log phase (2,000 cells of BxPC3 and 1,500 cells of Panc-1 per well) were seeded in 96-well plates. After a period of 24 hours, the cells were treated with concentrations ranging from 1.92 to 30,000.00 nmol/L for 72 hours. After this time, the survival fraction of the cells was determined. For this purpose, cell metabolism was used as a surrogate parameter for viability by using the CellTiter-Blue assay (Promega, Madison, USA). Normalization was based on untreated controls. Each individual measurement was carried out in triplicate and repeated three times.

### Statistical analysis

If not stated differently, all analyses were performed using R version 4.1. Responder stratification was performed using stratifyR (v1.0-3) with 2 strata and a fixed total sample size of 0.9 (29). Data handling was performed using the tidyverse package (v2.0.0) including ggplot2 for plotting.

## Results

### Morphology of single cell-derived cell lines (SCDCLs)

**Figure 1:**
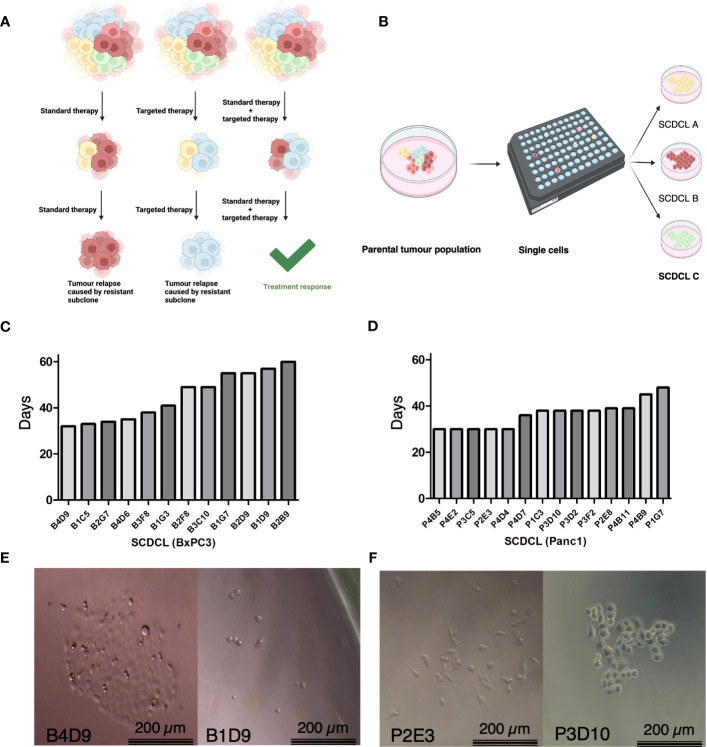
Schematic overview, growth kinetics of SCDCL and morphology. **(A)** Overview of intratumoral heterogeneity with subclones of diverse intrinsic resistance to the therapeutics. **(B)** Workflow for the establishment of SCDCLs from a parental tumor population by limited dilution. **(C)** Time of all 12 established SCDCLs of BxPC3 until 80% confluency in a 6-well culture plate ranging from 32 to 60 days. **(D)** Time of all 14 established SCDCLs of Panc-1 until 80% confluency in a 6-well culture plate ranging from 30 to 48 days. **(E)** Morphologic differences of initial colonies of BxPC3 at day 14 after single cell cloning. **(F)** Morphologic differences of initial colonies of Panc-1 at day 7 after single cell cloning.

We hypothesized that molecular preconditions of tumor cell subclones within heterogeneous pancreatic cancers correlate with differential response to therapy and could be targeted to modify treatment response (**Figure 1A**). Hence, we established single cell-derived cell lines (SCDCLs) of the parental cell populations from the classical differentiated cell line BxPC3 and basal-like cell line Panc-1 by limiting dilution. Twelve SCDCLs of BxPC3 and 14 SCDCLs of Panc-1 were generated as schematically shown in **Figure 1B**. The time period after single cell sorting until 80% confluency in a 6-well culture plate was considerably different between individual SCDCLs of both parental cell lines. Among the SCDCLs of BxPC3, the first one reached confluency after 32 days and the last one after 62 days (**Figure 1C**). The time to 80% confluency of the SCDCLs of Panc-1 ranged from 30 to 48 days (**Figure 1D**).

Cell morphology and growth patterns of the growing colonies differed between distinct SCDCLs of both cell lines as exemplified by the phase contrast images shown in **Figures 1E, F**. Although the BxPC3 SCDCLs all tended to grow in rather dense formations, we also observed a more elongated shape of the cells in some of the SCDCLs, while others grew much more cuboidal (**Figure 1E**). Notably, a higher number of spindle-shaped cells were observed in the SCDCLs of Panc-1 in some clonal cultures, indicating a more mesenchymal phenotype. Other SCDCLs of Panc-1, however, grew in more cobblestone-like formations and resemble more of a cuboid shape, which indicates a more epithelial phenotype, confirming earlier observations (**Figure 1F**) (17).

### SCDCLs respond differently to gemcitabine treatment

To test our central hypothesis, that different SCDCLs of the same parental tumor cell population of PDACs bear distinct intrinsic molecular profiles that determine the individual response to chemotherapy, each SCDCL was tested for its individual response to gemcitabine *in vitro*. First, we determined the half-maximal inhibitory concentration (IC_50_) and a concentration close to the maximal inhibitory concentration (IC_max_) of the BxPC3 and Panc-1 parental cell populations. Dose-response curves to gemcitabine were generated for both parental cell lines in the concentration range from 2.5 to 320 nmol/L. The parental Panc-1 population (IC_50 _= 43 nmol/l) proved to be much more resistant to gemcitabine than parental BxPC3 population (IC_50 _= 9.6 nmol/L) as measured by their respective IC_50_ (**Supplementary Information S1**).

**Figure 2:**
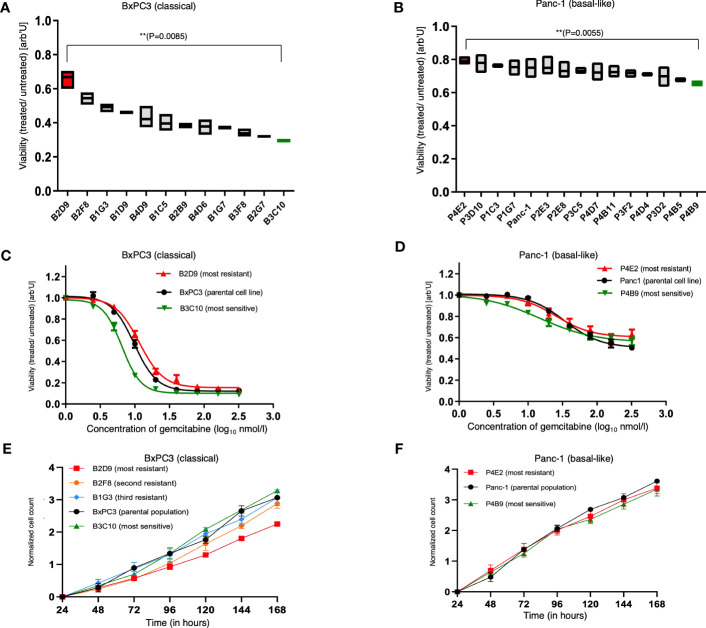
Treatment response and proliferation curves of the parental cell lines and SCDCLs. **(A)** Survival fraction of each SCDCL of BxPC3 in comparison to the control after treatment with 9.6 nmol/L (= IC_50_ of parental population) gemcitabine (mean ± min/max; ** p = 0.0085, unpaired t-test with Welch’s correction). **(B)** Survival fraction of each SCDCL of Panc-1 in comparison to the control after treatment with 43 nmol/L (= IC_50_ of parental population) gemcitabine (mean ± min/max; ** p = 0.0055, unpaired t-test with Welch’s correction). **(C)** Dose-response of the parental BxPC3 cell population, B2D9 (most resistant SCDCL to gemcitabine) and B3C10 (most sensitive SCDCL to gemcitabine) to gemcitabine (mean ± SEM). **(D)** Dose-response of the parental Panc-1 cell population, P4E2 (most resistant SCDCL to gemcitabine) and P4B9 (most sensitive SCDCL to gemcitabine) to gemcitabine (mean ± SEM). **(E)** Proliferation curves of the parental cell line BxPC3 and the SCDCLs B2D9, B2F8, B1G3 and B3C10 (mean ± SEM). **(F)** Proliferation curves of the parental cell line Panc-1 and the SCDCLs P4E2 and P4B9 (mean ± SEM).

Subsequently, each SCDCL of both parental cell populations was treated with the half-maximal inhibitory concentration (IC_50_) and a concentration close to the maximal inhibitory concentration (IC_max_) of the respective parental population. We observed a highly variable response of distinct SCDCLs of both cell lines BxPC3 and Panc-1 (**Figures 2A, B**; **Supplementary Information S2**). Heterogeneity in response was substantially higher among the BxPC3 SCDCLs compared to the Panc-1 SCDCLs.

In detail, the SCDCLs of BxPC3 showed a highly variable response. The most resistant SCDCL B2D9 had a survival rate of 0.67, while this was only 0.29 for the most sensitive SCDCL B3C10 when treated with IC_50_ of the parental cell population (p<0.009). There was a continuum of response rates to gemcitabine treatment between these extremes, although one might suspect a greater increase from the fourth most resistant SCDCL onwards (**Figure 2A**).

Among the SCDCLs of Panc-1, there was also a heterogeneous response, although the differences between the most resistant SCDCL P4E2 with a survival rate of 0.8 and the most sensitive SCDCL P4B9 with 0.66 (p<0.006), when treated with IC_50_ of the parental cell population, were considerably smaller. The SCDCLs with survival rates between these extremes formed a much denser continuum compared to BxPC3.

From the most sensitive and the most resistant SCDCL of both parental cell lines, complete dose-response curves were subsequently established, corresponding to the experimental design described above. These curves showed significant differences in the resistance profile between BxPC3 SCDCls B2D9 and B3C10 in concentration ranges from 5 nM to 80 nM (p<0.05) (**Figure 2C**). Among the Panc-1 SCDCLs P4E2 and P4B9, a significant difference was only observed at concentration ranges from 2.5 nM to 5nM (p<0.05) (**Figure 2D**).

Next, we also tested for the other SCDCLs whether the resistance to gemcitabine occurred randomly at the IC_50_ of the respective parental population, or rather indicated a more resistant behavior of the SCDCL in general. The response rates of the SCDCLs at the IC_50_ and IC_max_ tended to correlate for both cell lines (SCDCLs of BxPC3: r=0.4828; p<0.001; SCDCLs of Panc-1 r=0.3089; p<0.001) (**Supplementary Information S3**). Thus, we concluded that a higher resistance to gemcitabine at the IC_50_ of the respective parental population tends to reflect a higher resistance to gemcitabine of the respective SCDCL in general.

### Correlation between proliferative behavior and treatment response

Next, we tested whether there is a correlation between the time to confluency after single-cell sorting, which could be an indicator of better adaptive behavior and treatment response. For the SCDCLs of both cell lines, no clear association between the time to confluency and response to treatment was observed (**Supplementary Information S4**).

Gemcitabine, a deoxycytidine analog, causes inhibition of DNA chain elongation in addition to several other processes (30). To explore a potential correlation between proliferation rate and gemcitabine sensitivity, we determined population doubling times (PDT) of the most resistant SCDCLs, most sensitive SCDCLs and the parental tumor population of both cell lines. The most resistant SCDCL of BxPC3 (B2D9: PDT= 44.27h; CI95%: 41.29h to 47.72h) had a higher PDT in comparison to the most sensitive SCDCL (B3C10: PDT= 29.28h; CI95%: 26.99h to 32h) and the parental BxPC3 population (PDT: 31.47h; CI95%: 29.05h to 34.31h). For further validation, we additionally determined the PDT of the second and third most gemcitabine resistant SCDCL. However, PDTs of these SCDCLs were similar compared to the most sensitive SCDCL B3C10 and the parental cell line BxPC3 (B2F8; PDT= 34.13h; CI95%: 30.72h to 38.38h; B1G3: PDT= 32.82h; CI95%: 29.13h to 37.59h) (**Figure 2E**). There were no significant differences between the parental Panc-1 population (PDT= 26.86h CI95%: 25.18h to 28.79h), the most sensitive (P4B9: PDT= 29.91h; CI95%: 27.31h to 33.06h) and most resistant SCDCL (P4E2: PDT= 29.42h; CI95%: 26.33h to 33.34h) (**Figure 2F**).

We concluded that the observed differences in response to gemcitabine are a reflection of molecular idiosyncrasies of the individual SCDCLs that are independent of their intrinsic proliferation rates. Thus, we comprehensively profiled the transcriptome and proteome of individual SCDCLs of both parental cell populations to elucidate the molecular basis of the response of SCDCLs to gemcitabine.

### Transcriptomic differences between SCDCLs are associated with treatment response

To determine whether the distinct treatment phenotypes of the SCDCLs are related to transcriptomic heterogeneity, we performed mRNA sequencing of all 12 SCDCLs of BxPC3 and all 14 SCDCLs of Panc-1.

**Figure 3:**
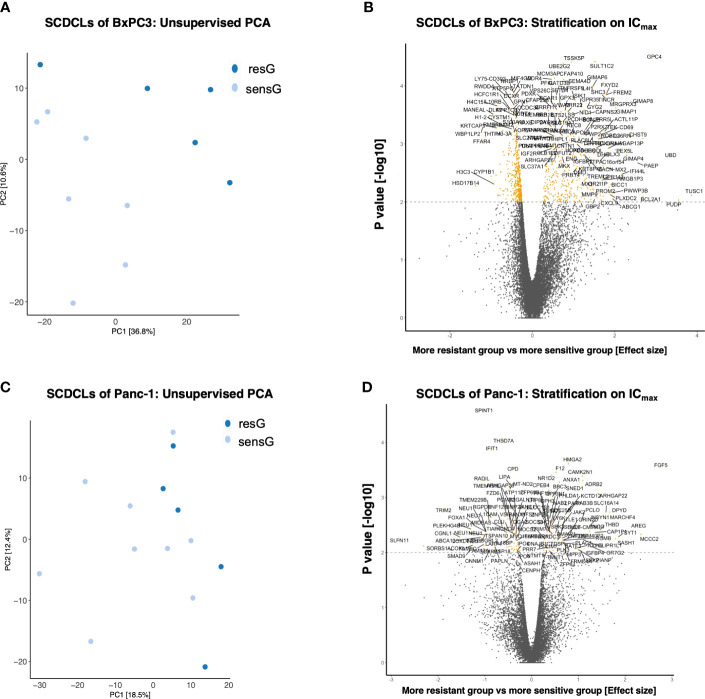
Transciptomics (mRNA-seq) of the SCDCLs of BxPC3 and Panc-1. **(A)** Unsupervised PCA including the 1,000 most variably expressed genes of the SCDCLs of BxPC3 **(B)** Stratification on IC_max;_ 753 genes were differentially expressed (q < 0.1; 327 up-regulated in resG, 426 up-regulated in sensG). **(C)** Unsupervised PCA including the 1,000 most variably expressed genes of the SCDCLs of Panc-1 **(D)** Stratification on IC_max;_ 149 genes differentially expressed (q < 0.1; 74 up-regulated in resG, 75 up-regulated in sensG).

Unsupervised Principle Component Analysis (PCA) on the 1,000 most variably expressed genes showed a clear clustering of the SCDCLs according to their respective sensitivity to gemcitabine treatment for BxPC3, while this clustering was less clear for Panc-1 (**Figures 3A, C**; **Supplementary Information 5A, C**).

For data evaluation, SCDCLs of each parental cell population were divided into two groups according to their respective treatment response, i.e., a more resistant group (resG) and a more sensitive group (sensG). The cut-off value of the survival rate for the assignment of each SCDCL to the respective group was calculated for treatment at the respective IC_50_ as well as IC_max_ according to Reddy et al. (29). For the SCDCLs of BxPC3, the cut-off values based on the IC_50_ and IC_max_ were 0.44 and 0.18, respectively. The cut-off values for Panc-1 were 0.73 and 0.49 based on the IC_50_ and IC_max_, respectively.

Next, we aimed to identify differentially expressed genes between SCDCLs of the resG compared to the sensG. When stratified according to the IC_50_ value, the SCDCLs of BxPC3 showed 159 differentially expressed genes (q < 0.1; 106 up-regulated in resG, 53 up-regulated in sensG) (**Supplementary Information 5B**). When stratified by IC_max_, 753 genes were differentially expressed (q < 0.1; 327 up-regulated in resG, 426 up-regulated in sensG) (**Figure 3B**). Consistent with less heterogeneity in response to gemcitabine, Panc-1 SCDCLs showed fewer differentially expressed genes. When stratified by IC_50_ value 98 genes were differentially expressed (q < 0.1; 37 up-regulated in resG, 61 up-regulated in sensG) (**Supplementary Information 5D**), and when stratified by IC_max_ 149 genes were differentially expressed (q < 0.1; 74 up-regulated in resG, 75 up-regulated in sensG) (**Figure 3D**). Measured by the number of differentially expressed genes in the SCDCLs of the respective parental cell populations, we conclude that there is less transcriptional heterogeneity between SCDCLs of the basal-like cell line Panc-1 compared to the SCDCLs of the classical cell line BxPC3. Strikingly, the lower transcriptional heterogeneity is reflected in the lower heterogeneity of response to gemcitabine treatment.

### SCDCL transcriptomes reveal resistance-associated pathway enrichment

**Figure 4:**
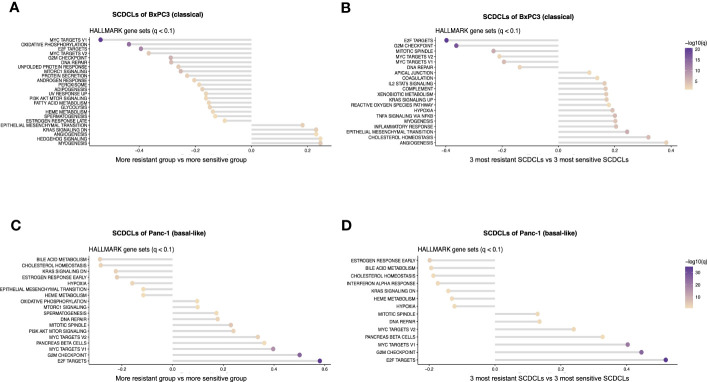
Pathway enrichment analysis of the SCDCLs of BxPC3 and Panc-1. **(A)** Graphical overview of pathway enrichment of resG versus sensG in SCDCLs of BxPC3. **(B)** Graphical overview of pathway enrichment of the three most resistant versus the three most sensitive SCDCLs of BxPC3. **(C)** Graphical overview of pathway enrichment of resG versus sensG in SCDCLs of Panc-1. **(D)** Graphical overview of pathway enrichment of the three most resistant versus the three most sensitive SCDCLs of Panc-1.

Next, gene set enrichment analysis was performed to identify the differentially regulated signaling pathways between the resG and the sensG (**Figures 4A, C**).

The SCDCLs of both parental cell populations showed a continuum between the most resistant and most sensitive SCDCL in their response to gemcitabine (**Figures 2A, B**). Hence, we hypothesized a rather continuous differential regulation of relevant signaling pathways rather than on-off effects. We performed additional GSEA between the three most resistant SCDCLs (BxPC3: B2D9, B2F8, B1G3; Panc-1: P4E2, P3D10, P1C3) and the three most sensitive SCDCLs (BxPC3: B3C10, B2G7, B3F8; Panc-1: P4B9, P4B5, P3D2) of both, BxPC3 and Panc-1, to be able to generate a better discriminatory power of differentially regulated pathways (**Figures 4B, D**).

The pathways MYC targets V1, MYC targets V2 as well as E2F targets from the Molecular Signatures Database (MSigDB) were enriched in the sensitive SCDCL of BxPC3 across all comparisons. Interestingly, exactly these pathways were enriched in Panc-1 in the group of the resistant SCDCLs across all comparisons. In contrast, enrichment of EMT correlated with resistance to gemcitabine in BxPC3 SCDCLs across all comparisons, while Panc-1 SCDCLs showed no resistance-associated enrichment of this pathway.

As described above, we hypothesize that relevant signaling pathways are gradually differentially regulated in the SCDCLs. Hence, particular attention was paid to pathways that did not show significant differential expression in the resG and sensG comparisons, but in contrast, were differentially regulated in the comparison of the three most resistant versus the three most sensitive SCDCLs. Since a positive correlation was observed between the response rates of the SCDCLs to therapy at the IC_50_ (**Figures 4A–D**) as well as the response rate at the IC_max_ (**Supplementary Information S6A–D**), the differentially enriched pathways overlapping in the different comparisons were further considered. Overlaps of enriched gene sets between all comparisons are shown in a tabular overview in **Supplementary Information S6E, F**. Consequently, in BxPC3 SCDCLs, enriched signaling pathways EMT, TNF signaling via NfKB, and IL2STAT5 signaling correlated with more resistant behavior to gemcitabine. In addition, several other inflammatory signaling pathways appear to be associated with resistance. An overview of the differentially regulated pathways according to IC_max_ classification is shown in **Supplementary Information 6**. In contrast to BxPC3, no additional enriched pathways were found in the SCDCLs of Panc-1, when comparing the three most resistant and the three most sensitive SCDCLs. This result is in line with the lower transcriptional heterogeneity among SCDCLs of Panc-1.

### SCDCL reveals resistance-associated protein signatures

For a comprehensive understanding of the biological processes associated with the heterogeneity of SCDCLs and their distinct intrinsic resistance profiles to gemcitabine, we next sought to profile their individual proteomes. As commonly known, mRNA expression levels do not necessarily reflect the respective protein expression levels (31). To obtain a more comprehensive picture, we therefore aimed to identify proteins that were associated with the heterogeneous response to gemcitabine of individual SCDCLs by mass spectrometry analyses. Using the feature selection with the random forest approach, we extracted protein signatures that are associated with the response to gemcitabine of each individual SCDCL. Extracted proteins were ranked according to their predictive value for treatment response. This approach reflects the fact that different protein compositions may be similarly important for adaptation to the IC_50_ target variable

**Figure 5:**
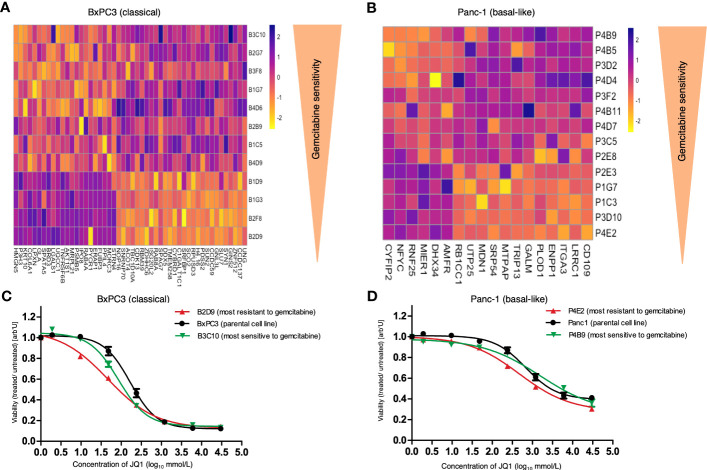
Heatmap of extracted proteins associated with response to gemcitabine and response of parental cell lines and SCDCLs to JQ1. **(A)** Heatmap of extracted proteins associated with the functional heterogeneity of response to gemcitabine of individual SCDCLs in BxPC3. Twenty-one proteins were associated with poorer and 29 proteins with a better response to gemcitabine treatment. **(B)** Heatmap of extracted proteins associated with the functional heterogeneity of response to gemcitabine of individual SCDCLs in Panc-1. Six proteins were associated with poorer and 12 proteins with a better response to gemcitabine treatment. **(C)** Dose response of the parental BxPC3 cell population, B2D9 (most resistant SCDCL to gemcitabine) and B3C10 (most sensitive SCDCL to gemcitabine) to JQ1 (mean ± SEM). **(D)** Dose response of the parental Panc-1 cell population, P4E2 (most resistant SCDCL to gemcitabine) and P4B9 (most sensitive SCDCL to gemcitabine) to JQ1 (mean ± SEM).

For BxPC3, we extracted 50 proteins that were associated with the functional heterogeneity of response to gemcitabine of individual SCDCLs (**Supplementary Information S7**). Of these, overexpression of 21 proteins was associated with a poorer response to gemcitabine, whereas overexpression of 29 proteins was associated with a better response to gemcitabine (**Figure 5A**). TNF receptor superfamily member 6b (TNFRSF6B) was ranked highest among the proteins extracted for BxPC3 and associated with a poorer response. In addition, DNA activity-influencing proteins such as bromodomain 3 (BRD3) and high mobility group nucleosome binding domain 5 (HMGN5) were highly ranked in the identified set of proteins associated with a poorer response. Among others, HMGN5 was also expressed significantly stronger at the mRNA level in the resG compared to the sensG (p=0.046).

For Panc-1, we again identified substantially fewer proteins whose expression level was associated with response to treatment of individual SCDCLs which is in line with the lower transcriptional heterogeneity described above. A set of 18 proteins was identified that was associated with the heterogeneity of response to gemcitabine of individual SCDCLs (**Supplementary Information S7**). Of these, overexpression of six proteins was associated with a poorer response to gemcitabine, whereas overexpression of 12 proteins was associated with a better response to gemcitabine (**Figure 5B**). Autocrine Motility Factor Receptor (AMFR) was ranked highest among the proteins extracted for Panc-1 (**Supplementary Information S7**).

### Gemcitabine-resistant SCDCLs are more sensitive to JQ1

JQ1 is an inhibitor of the bromodomain and extraterminal family of proteins (BET) with the highest selectivity for BRD4 (32).

In our pathway enrichment analyses based on transcriptomics described above, the more gemcitabine-resistant SCDCLs of the classical cell line BxPC3 showed enrichment of EMT, TNF signaling via NfKB, and IL2STAT5 signaling. The proteome analyses of the same SCDCLs identified two pivotal proteins in the extracted protein signature, i.e., (i) BRD3 which is a family member of the BET proteins and (ii) the TNF receptor TNFRSF6B whose gene possess a super-enhancer in multiple myeloma cells (33).

Pathway enrichment analyses based on transcriptomics of the gemcitabine-resistant SCDCLs of the basal-like cell line Panc-1 showed enrichment of MYC signaling, i.e., gene sets MYC targets V1 and MYC targets V2.

JQ1 suppresses cell proliferation through several signaling pathways such as TNFA_Signaling_via_nfkb, L2_STAT5_SIGNALING, MYC signaling as well as multiple inflammatory transcriptional programs in pancreatic cancer (33–37).

As described above, we intended to identify molecular preconditions of tumor cell subclones that could be targeted to overcome treatment resistance of current clinical standard therapy (**Figure 1A**). In our model system, these subclones are reflected by SCDCLs of heterogeneous parental cell populations which show differential response to treatment with gemcitabine. Based on our molecular findings, we hypothesize that gemcitabine-resistant SCDCLs could be specifically targeted by inhibition of proteins of the BET family in both, the classical cell line BxPC3 and the basal-like cell line Panc-1. Therefore, we tested the specific anti-proliferative effect of JQ1, which is an effective inhibitor of the BET proteins, in our SCDCL model system (32).

In general, the parental population of Panc-1 (IC_50_: 679 nM CI95%: 496.5 – 929.1) was more resistant to JQ1 treatment compared to the parental BxPC3 population (IC_50_: 184.3 nM; CI95%: 146.3 - 232.2). Next, we generated dose-response curves for JQ1 of the parental cell population, the most gemctabine-resistant SCDCL and the most gemcitabine-sensitive SCDCL for both cell lines, BxPC3 and Panc-1.

In BxPC3 the most gemcitabine-resistant SCDCL B2D9 was the substantially more sensitive to treatment with JQ1 (IC_50_: 48.36 nM; CI95%: 27.68 - 84.48). compared to the most gemcitabine-sensitive SCDCL B3C10 (IC_50_: 95.63 nM CI95%: 73.16 - 125) (**Figure 5C**). Differential response was significant in concentration ranges from 9.6 nM to 48 nM (p<0.02). The parental BxPC3 population was significantly more resistant to JQ1 treatment (IC_50_: 184.3 nM; CI95%: 146.3 - 232.2) compared to both derived SCDCLs.

The most gemcitabine-resistant SCDCL of Panc-1 again was most sensitive when treated with JQ1 (P4E2: IC_50_: 471.8 nM CI95%: 338.4 – 658) in comparison to the most gemcitabine-sensitive SCDCL P4B9 (IC_50_: 1590 nM CI95%: 667 – 3791). The differential response was significant in concentration ranges from 240 nM to 6000 nM (p<0.02). The parental population Panc-1 was in-between these two SCDCLs (IC_50_: 679 nM CI95%: 496.5 – 929.1) (**Figure 5D**).

In conclusion, we showed that gemcitabine-resistant subclones, i.e., SCDCLs, of the heterogeneous parental cell populations of both the classical cell line BxPC3 and the basal-like cell line Panc-1 can be specifically targeted using the BET inhibitor JQ1.

## Discussion

In recent years technical advances such as single-cell RNA sequencing or barcoding technologies have developed experimental methods that can unveil an ever-increasing extent of ITH (38, 39). Single-cell RNA analyses showed that cells of the basal-like subtype are much more widespread than generally assumed and can also be detected in classical classified pancreatic cancers (10). Moreover, recent studies described single cells expressing both classical and basal markers (11, 40). Such co-expressing cells appear to reflect an intermediately differentiated state. Thus, intertumoral subtyping alone is not fully reflecting the complex tumor biology of heterogeneous PDACs. The fact that higher levels of ITH correlate with shorter patient survival underscores its significance (11). There is evidence that resistant subclones are already present in small populations of tumor cells prior to initiation of therapy which results in treatment failure (9, 16, 41). A deeper understanding of intratumoral heterogeneity and these resistant subpopulations could therefore help to overcome therapy resistance and tumor relapse.

We recently described SCDCLs derived from single cells as an *ex vivo* model to decipher functional differences in expression profiles and therapy response among individual clones from primary rectal tumors (42). In the present study, we generated a total of 26 SCDCLs from the well-established PDAC classical cell line BxPC3 and basal-like cell line Panc-1. We acknowledge that our approach provides only a snapshot of the ITH of pancreatic tumors in real world. By the present study based on SCDCLs we did not attempt, nor would it be feasible, to reconstruct the complete complex clonal architecture of pancreatic cancers. However, our approach provides a model that allows to analyze heterogeneity from a functional point of view.

Yachida et al. showed that subclones forming distant metastases are present within the primary tumor and arise from the non-metastaic parental population. These clones may develop long before the metastatic event (43). For both cell lines BxPC3 and Panc-1 we observed substantially different length of time to confluency after single cell cloning which might reflect different adaptive abilities of distinct clones to new environments. In a recent study, we proved that SCDCLs of the basal-like cell line Panc-1 had different epithelial/mesenchymal phenotypes, differed in their invasive behavior, and therefore exhibited different tumorigenic potential *in vitro* (17).

The aim of this study was to explore the potential heterogeneity of response to gemcitabine, as a clinical standard treatment regimen, in the pancreatic cancer cell lines BxPC3 and Panc-1. We subsequently aimed to identify gemcitabine-resistant subclones and to identify potential molecular targets for improved therapy of these subclones.

We observed a highly variable response to gemcitabine of distinct SCDCLs of both cell lines (**Figures 2A, B**). Heterogeneity in response was substantially higher among the BxPC3 SCDCLs compared to the Panc-1 SCDCLs. There was a continuum of response rates to gemcitabine treatment between the most resistant and most sensitive SCDCLs of both parental cell populations. However, the SCDCLs derived from Panc-1 formed a much denser continuum compared to BxPC3 reflecting a lower heterogeneity in gemcitabine-response in Panc-1. The dose-response curve of the parental population Panc-1 showed a much less steep inflection point at the IC_50_ than that of the BxPC3 population and might result in better discrimination of treatment response for BxPC3 when treated at the IC_50_. Conversely, our analysis of the transcriptome revealed less transcriptional differences in the SCDLCs of Panc-1, suggesting a generally less intratumoral heterogeneity of this basal-like cell line. In summary, it can be stated for both cell lines that there is no clear cut-off between resistance and sensitivity, as the SCDCLs form a continuum across response rates. Indeed, we could show the same distinct response to gemcitabine of individual SCDCLs that were derived from our primary patient-derived pancreatic cancer cell line LuPanc-1 (41 and data unpublished).

After demonstrating distinct intrinsic behavior of individual SCDCLs of both parental cell populations in terms of response/resistance to gemcitabine treatment, we performed transcriptomic analysis. For a comprehensive understanding of the biological processes associated with the heterogeneity of SCDCLs, GSEA with Hallmark Gene Sets was performed. With this, we ultimately aimed to identify potential molecular targets which might help to modify therapy to especially target gemcitabine-resistant subclones.

Among the more resistant group of SCDCLs of Panc-1, we observed differentially enriched signaling pathways, i.e., (i) MYC targets V1, (ii) MYC targets V2, (iii) G2M checkpoints, and (iv) E2F targets. Indeed, low MYC RNA levels are associated with sensitivity to gemcitabine and c-MYC overexpression correlates with gemcitabine resistance (44, 45). Upregulated G2M checkpoint signaling is associated with impaired survival of pancreatic cancer patients (46). Published literature for E2F targets, however, is contradictory. On the one hand, E2F target expression seems to be related to impaired clinical outcome and is also predictive of response to E2F inhibitors in *in vitro* experiments, but not of response to gemcitabine or other chemotherapy-based treatments in pancreatic cancer (47). On the other hand, further studies on different tumor entities showed an association between E2F-1 and resistance to chemotherapy (48–50). In our current study, E2F pathway was associated with gemcitabine resistance in the SCDCLs of Panc-1, whereas the opposite was true in the SCDCLs of BxPC3. One might speculate that the effect of E2F signaling in terms of treatment response is associated with the molecular subtype, i.e., classical (BxPC3) or basal-like (Panc-1).

Strikingly, also MYC targets V1, MYC targets V2, and the G2M checkpoint pathway that were enriched in the gemcitabine-resistant SCDCLs of Panc-1, were enriched in the gemcitabine-sensitive SCDCLs of BxPC3. Whether this is due to a hierarchical functional relevance of these pathways with respect to resistance to gemcitabine or whether this is due to the different molecular subtypes of the two parental cell lines can only be speculated at this point.

In the resistant SCDCLs of BxPC3, we observed enrichment of numerous signaling pathways such as (i) EMT, (ii) TNFA via NfkB, and (iii) IL2STAT5. It is well known that EMT in pancreatic cancer cells contributes to gemcitabine resistance and decreases overall survival in mouse models (51). In addition, there is evidence that several EMT regulators induce drug resistance in human pancreatic cancer (52). NfkB signaling has been described to be constitutively active in a large proportion of pancreatic tumors and high basal levels of this transcription factor appear to play an important role in mediating chemotherapy resistance (53–55). Moreover, gemcitabine treatment can induce activation of NfkB and STAT3 in pancreatic cancer and can thereby induce resistance to itself (56). The signal transducer and activator of transcription STAT5 affects several oncogenes and plays a role in crucial functions such as cell proliferation, apoptosis and cell differentiation (57, 58).

As it is generally accepted that mRNA expression levels do not necessarily reflect the respective protein expression levels (31), we additionally aimed to identify protein signatures that are associated with the heterogeneous response to gemcitabine.

We identified protein signatures for both BxPC3 and Panc-1 SCDCLs using a machine-learning approach, which were associated with the treatment response of individual SCDCLs. We subsequently extracted individual proteins that were associated with the signaling pathways that were enriched in the transcriptomic analyses. For BxPC3 (i) the TNF receptor TNFRSF6b, (ii) the nuclear protein HMGN5, and (iii) the BET protein BRD3 were extracted among others.

In both colon and gastric cancers, TNFRSF6b induces EMT via various signaling pathways and affects the growth and metastasis potential in colon carcinoma (59–61). In PDAC, TNFRSF6b also promotes proliferation and tumor growth and is associated with worse outcomes (62). HMGN5 (NSBP1) is a member of the HMGN nucleosome-binding protein family and, through its interaction with DNA, affects the architecture of chromatin and thus the transcriptome profile (63). It contributes to chemotherapy resistance in various tumor types such as osteosarcomas, squamous cell carcinomas of the esophagus, and germ cell tumors of the testes (64–66). However, its specific role in pancreatic cancers remains to be elucidated.

We identified BET proteins as potential targets in the gemcitabine-resistant SCDCLs of both cell lines BxPC3 and Panc-1. In fact, previous studies showed a synergistic effect of combined therapy of PDAC cells *in vitro* with gemcitabine and BET inhibitors (67, 68).

The BET inhibitor JQ1 affects expression of several gene targets with greatest selectivity for BRD4 (32). This ultimately leads to a depletion of the BET proteins from DNA, which affects the transcription of genes, especially genes with so-called super-enhancers (33, 69).

SCDCL-specific treatment with JQ1 revealed that both gemcitabine-most-resistant SCDCLs appeared to be more sensitive than the parental cell population and the gemcitabine-most-sensitive SCDCLs.

For c-MYC, it has already been shown in PDAC and other tumor entities that inhibition of BET proteins reduces the transcription of c-MYC and causes growth inhibition (70–72). As MYC signaling was enriched in the gemcitabine-resistant Panc-1 SCDCLs, the better response to JQ1 treatment in comparison to the gemcitabine-sensitive SCDCL might be caused by the higher sensitivity to reduced c-MYC transcription in Panc-1.

Strikingly, enrichment in MYC signaling was associated with gemcitabine-sensitivity in SCDCLs of BxPC3. In line, the gemcitabine-sensitive SCDCL of BxPC3 responded well to treatment with JQ1, albeit the response of the gemcitabine-resistant SCDCL was even better. At the first glance, this finding seems to be contradictory. However, as described above JQ1 treatment affects several signaling pathways besides MYC signaling. TNFRSF6b has been described to possess a super-enhancer that is occupied by BRD4 (33) and is overexpressed in the gemcitabine-resistant SCDCLs of BxPC3. Thus, JQ1 potentially inhibits transcription of TNFRSF6b and might thereby result in higher sensitivity of the gemcitabine-resistant SCDCL compared to the gemcitabine-sensitive SCDCL in BxPC3. In addition to TNFRSF6b overexpression, TNFA via NfkB, and IL2STAT5 signaling was also associated with gemcitabine resistance in SCDCLs of BxPC3. The relA subunit of NfkB binds to BRD4 via acetylated lysine-310, protecting it from degradation and stimulating the transcriptional activity of NfkB. Inhibition of BRD4 by JQ1 results in reduced nuclear levels of NfkB and therefore reduced TNFA-induced NfkB target gene expression (37). As a potential third mechanism, JQ1 removes BRD2 from chromatin and subsequently inhibits STAT5 and the expression of its target genes (73).

We acknowledge that our current study does not elucidate specific molecular mechanisms of JQ1 treatment in specific SCDCLs and needs further experimental in-depth studies. As discussed above, several distinct signaling pathways might mediate JQ1 effects. However, the focus of our current study was to uncover the heterogeneity of treatment response of distinct tumor cell subpopulations in an *in vitro* model and uncover the molecular preconditions of those subpopulations. Using SCDCLs of the classical cell line BxPC3 and the basal-like cell line Panc1, we showed considerable heterogeneity of response to gemcitabine which was based on distinct molecular preconditions. Our present study shows that understanding subclonal resistance mechanisms in heterogeneous tumor cell populations of PDACs might ultimately help to develop new treatment strategies as exemplified by JQ1 treatment. Pishvaian et al. highlighted that pancreatic cancer patients who received molecularly guided therapy compared to patients who received standard therapy had a significantly better survival (74). Our study underlines that especially treatment of heterogeneous pancreatic cancers requires individual patient-specific molecularly guided (combination) treatment strategies rather than a “one-size-fits-all” approach.

## Data availability statement

The datasets presented in this study can be found in online repositories. The names of the repository/repositories and accession number(s) can be found below: https://www.ncbi.nlm.nih.gov/, GSE232549; http://www.proteomexchange.org/, PXD042256.

## Ethics statement

Ethical approval was not required for the studies on humans in accordance with the local legislation and institutional requirements because only commercially available established cell lines were used.

## Author contributions

Conception and design: RB and BF. Development of methodology: BF, MK, and AK. Acquisition of data: BF, OL, KB, and JW. Analysis and interpretation of data: BF, RB, AK, MK, TS, MtW, and HU. Writing, review and/or revision of the manuscript: BF, RB, KH, LB, AK, BH, HB, UW, TK, TG, and HU. Administrative, technical, or material support: HB, TG, and TK. Study supervision: RB. All authors contributed to the article and approved the submitted version.

## Supplementary material

Supplementary Information S1 Treatment response of parental cell lines BxPC3 (classical) and Panc-1 (basal-like) to gemcitabine. Data are the means ± SEM of three independent experiments. **(A)** Dose-response of the parental BxPC3 cell population to gemcitabine. **(B)** Dose-response of the parental Panc-1 cell population to gemcitabine.

Supplementary Information S2 Treatment response of the parental cell lines and SCDCLs to gemcitabine. Data are means ± min. to max. **(A)** Survival fraction of each SCDCL of BxPC3 in comparison to the control after treatment with 160 nmol/L (= IC_max_ of parental population) gemcitabine. **(B)** Survival fraction of each SCDCL of Panc1 in comparison to the control after treatment with 160 nmol/L (= IC_max_ of parental population) gemcitabine.

Supplementary Information S3 Linear regression of treatment with the IC_50_ concentration of gemcitabine of the respective parental population and IC_max_. Data are the means ± SEM. **(A)** Treatment response of each SCDCLs of BxPC3 to IC_50_ (9,6nM) of the parental population against treatment response to IC_max_ (160nM). **(B)** Treatment response of each SCDCLs of Panc-1 to IC_50_ (43 nM) of the parental population against treatment response to IC_max_ (160nM).

Supplementary Information S4 Survival Fraction of each subclone after treatment with the IC_50_ concentration of gemcitabine of the respective parental population or IC_max_ (160nM) against time to confluency in a 6-well plate. Data are the means ± SEM of three independent experiments. **(A)** Treatment response of each SCDCLs of BxPC3 to IC_50_ (9,6nM) of the parental population against time to confluency in a 6-well. **(B)** Treatment response of each SCDCLs of BxPC3 to IC_max_ (160nM) against time to confluency in a 6-well. **(C)** Treatment response of each SCDCLs of Panc-1 to IC_50_ (43nM) of the parental population against time to confluency in a 6-well. **D,** Treatment response of each SCDCLs of Panc-1 to IC_max_ (160nM) of the parental population against time to confluency in a 6-well.

Supplementary Information S5 Transcriptomics (mRNA-seq) of SCDCLs of BxPC3 and Panc-1. **(A)** Unsupervised PCA including the 1,000 most variably expressed genes of the SCDCLs of BxPC3. (**B)** Stratification on IC_50_; 159 genes differentially expressed (q < 0.1; 106 up-regulated in resG, 53 up-regulated in sensG). **(C)** Unsupervised PCA including the 1,000 most variably expressed genes of the SCDCLs of Panc-1. **(D)** Stratification on IC_50_; 98 genes were differentially expressed (q < 0.1; 37 up-regulated in resG, 61 up-regulated in sensG).

Supplementary Information S6 mRNA-seq and pathway enrichment analysis of the parental cell population and SCDCLs of BxPC3 and Panc-1. (**A)** Graphical overview of pathway enrichment of resG versus sensG in SCDCLs of BxPC3 by grouping on the basis of the IC_max_ responses. **(B)** Graphical overview of pathway enrichment of the three most resistant versus the three most sensitive SCDCLs of BxPC3 by grouping based on the IC_max_ responses. **(C)** Graphical overview of pathway enrichment of resG versus sensG in SCDCLs of Panc-1 by grouping based on the IC_max_ responses. **(D)** Graphical overview of pathway enrichment of the three most resistant versus the three most sensitive SCDCLs of Panc1 by grouping based on the IC_max_ responses. **(E)** Overview of all gene set enrichment analyses of the SCDCLs of BxPC3. Pathways marked in grey were significantly enriched in the more resistant group/3 most resistant SCDCLs, respectively. **(F)** Overview of all gene set enrichment analyses of the SCDCLs of Panc-1. Pathways marked in grey were significantly enriched in the more resistant group/3 most resistant SCDCLs, respectively.
