# Supplementary figures and images for: Molecular profiling and specific targeting of gemcitabine-resistant subclones in heterogeneous pancreatic cancer cell populations
Source: Front Oncol. 2023 Aug 31;13:1230382. doi: 10.3389/fonc.2023.1230382 (PMC10502231; doi:10.3389/fonc.2023.1230382)

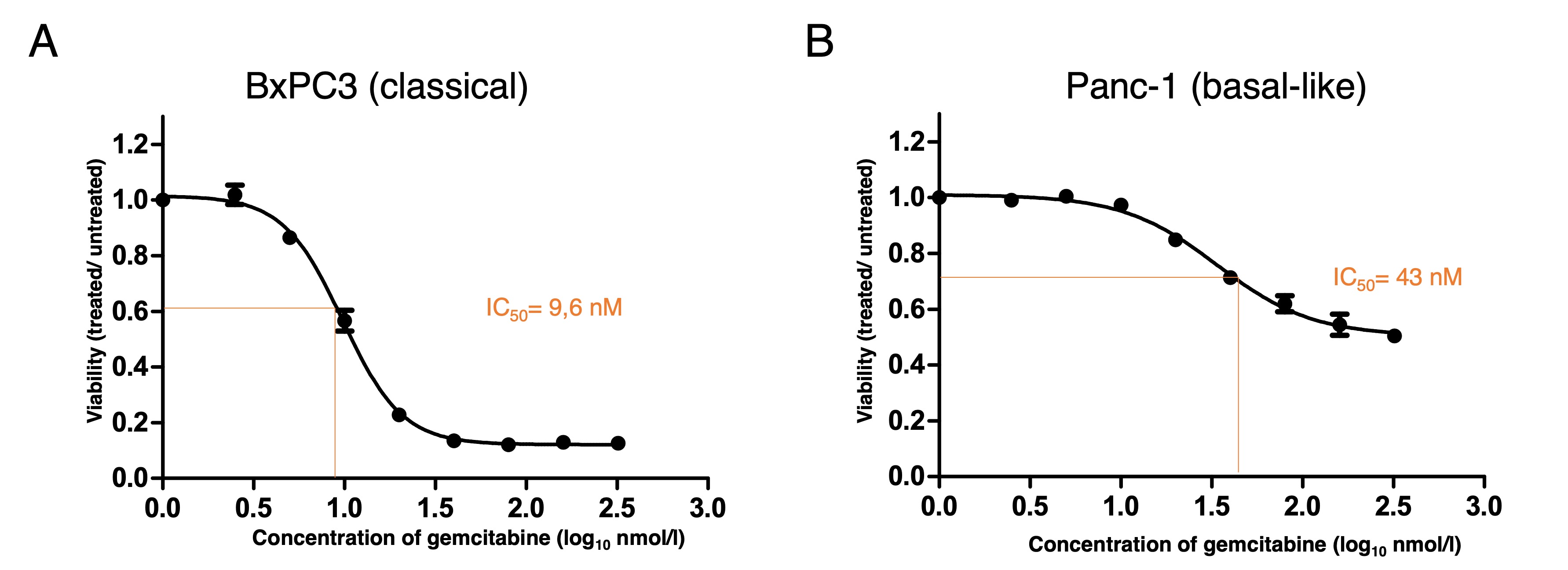

Supplement: Supplementary file 2 [file Image_1.jpeg]

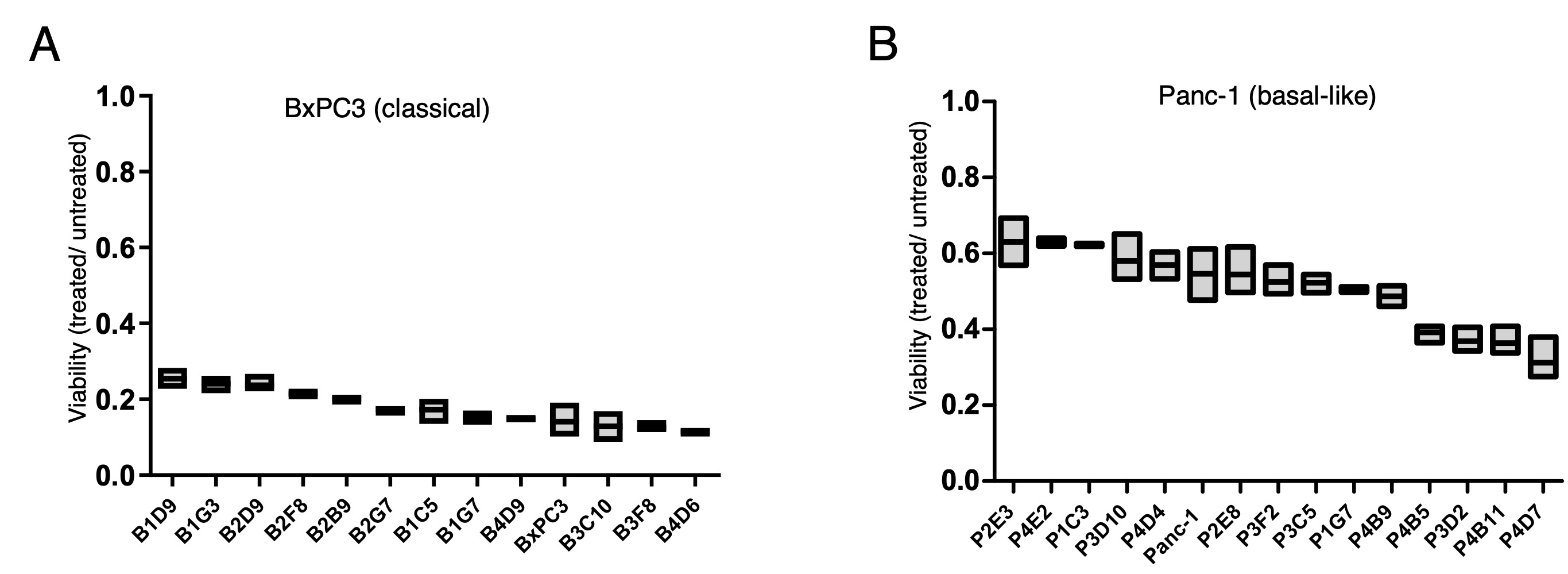

Supplement: Supplementary file 3 [file Image_2.jpeg]

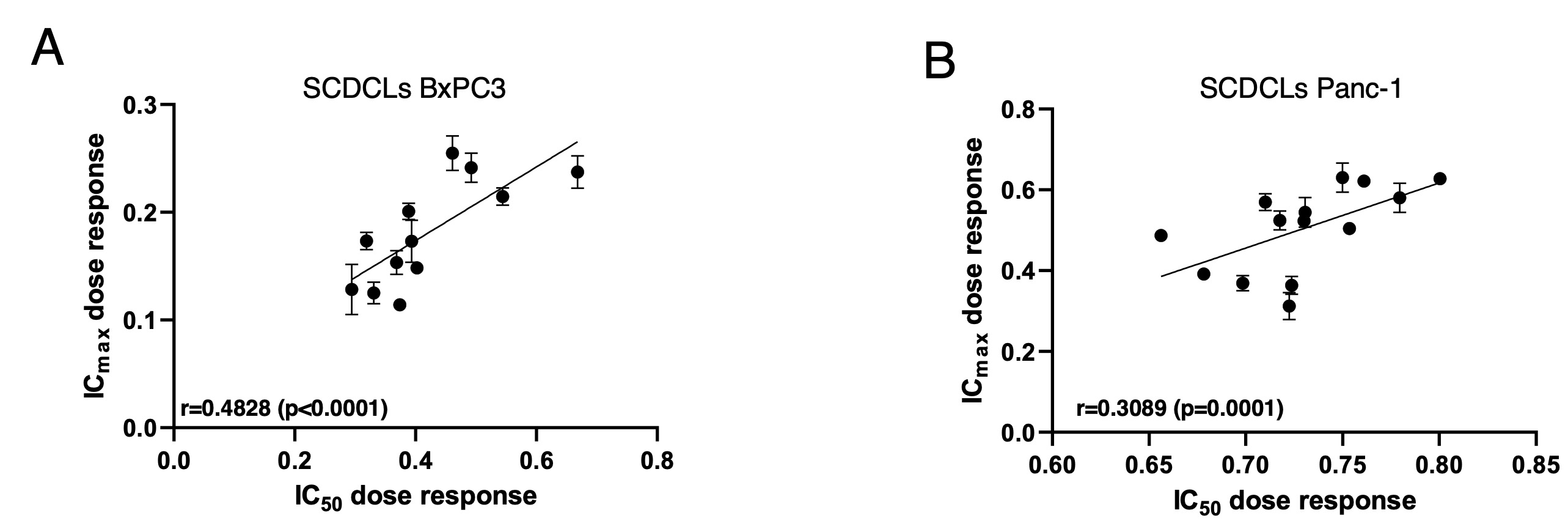

Supplement: Supplementary file 4 [file Image_3.jpeg]

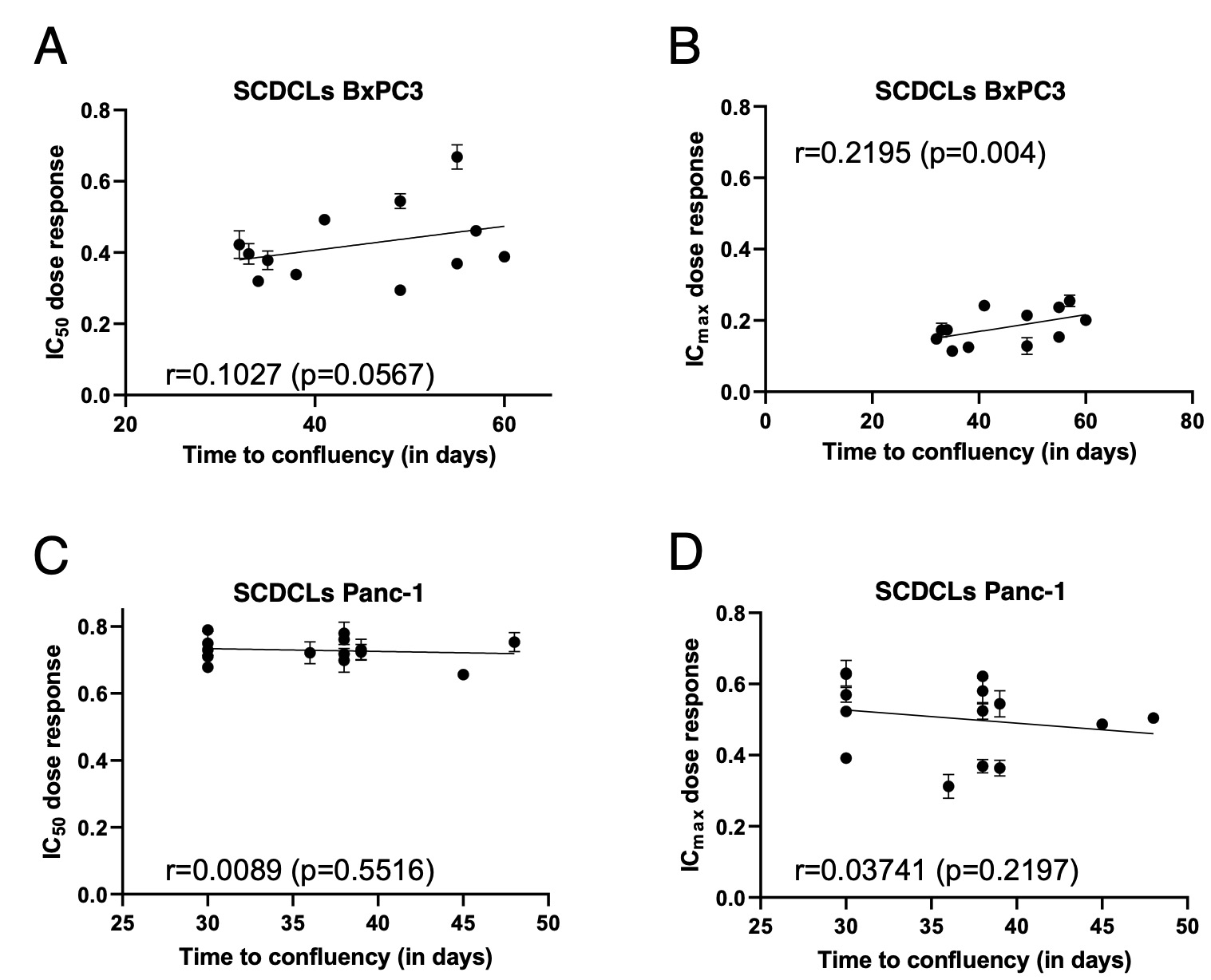

Supplement: Supplementary file 5 [file Image_4.jpeg]

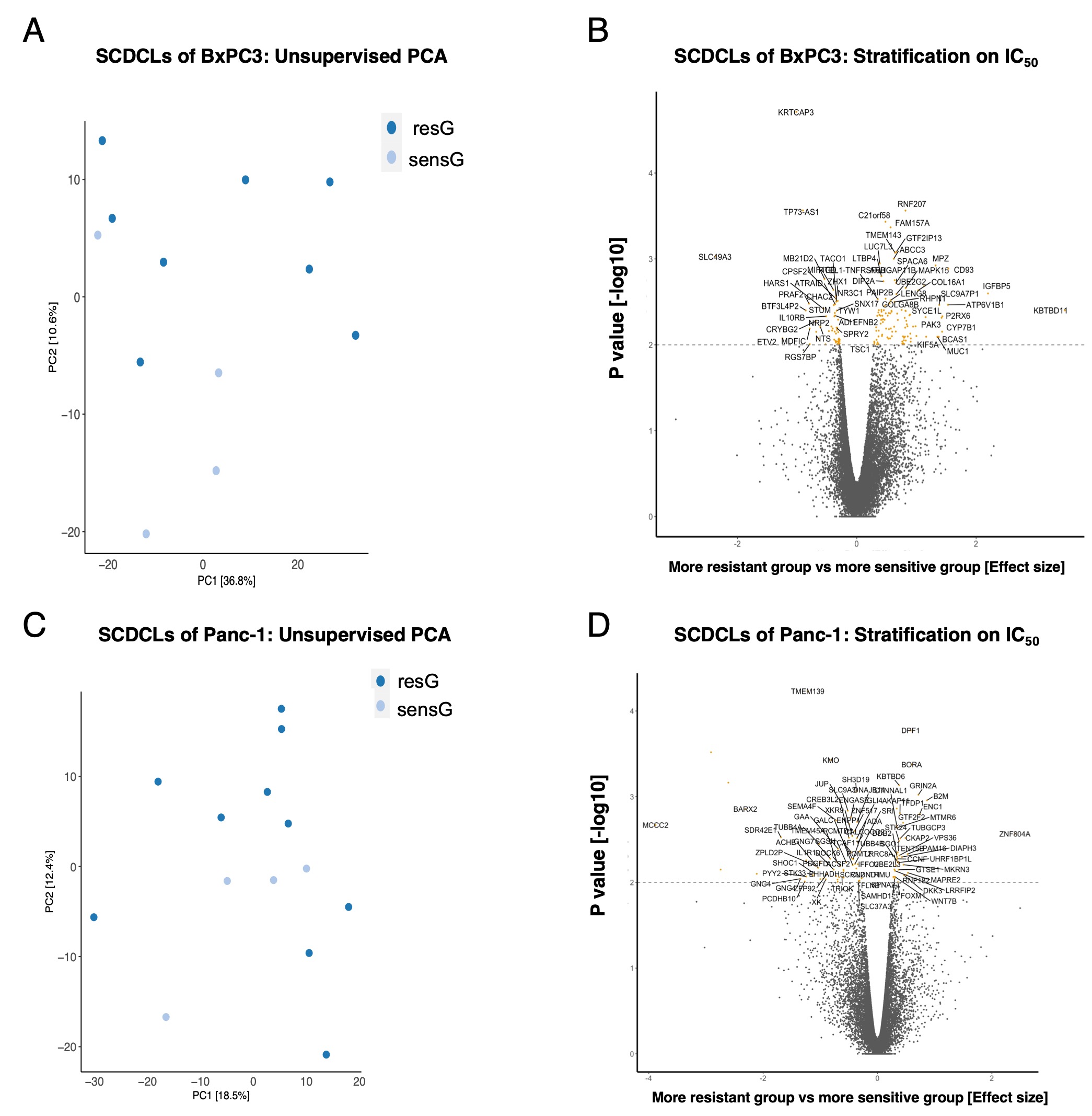

Supplement: Supplementary file 6 [file Image_5.jpeg]

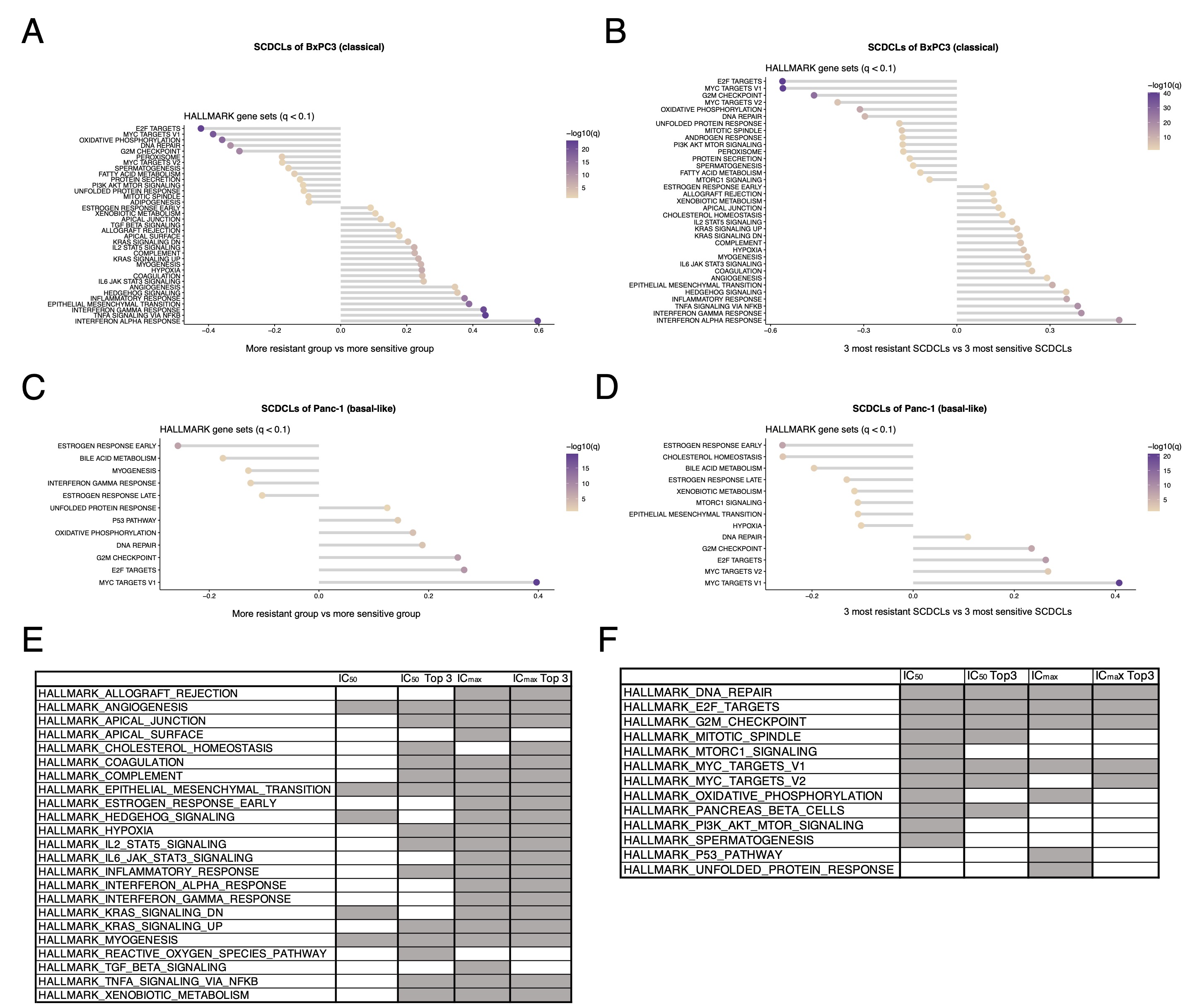

Supplement: Supplementary file 7 [file Image_6.jpeg]
